# Low-Dose Imaging in a New Preclinical Total-Body PET/CT Scanner

**DOI:** 10.3389/fmed.2019.00088

**Published:** 2019-05-03

**Authors:** Cesar Molinos, Todd Sasser, Phil Salmon, Willy Gsell, David Viertl, James C. Massey, Krzysztof Mińczuk, Jie Li, Bijoy K. Kundu, Stuart Berr, Carlos Correcher, Ali Bahadur, Ali A. Attarwala, Simon Stark, Sven Junge, Uwe Himmelreich, John O. Prior, Kjell Laperre, Sonica Van Wyk, Michael Heidenreich

**Affiliations:** ^1^Bruker BioSpin, Preclinical Imaging, Ettlingen, Germany; ^2^MoSAIC, KU Leuven, Leuven, Belgium; ^3^Department of Nuclear Medicine and Molecular Imaging, Lausanne University Hospital, Lausanne, Switzerland; ^4^Department of Radiology and Medical Imaging, University of Virginia, Charlottesville, VA, United States

**Keywords:** preclinical, imaging, PET, CT, low dose, [^18^F]FDG, oncology, total-body

## Abstract

Ionizing radiation constitutes a health risk to imaging scientists and study animals. Both PET and CT produce ionizing radiation. CT doses in pre-clinical *in vivo* imaging typically range from 50 to 1,000 mGy and biological effects in mice at this dose range have been previously described. [^18^F]FDG body doses in mice have been estimated to be in the range of 100 mGy for [^18^F]FDG. Yearly, the average whole body doses due to handling of activity by PET technologists are reported to be 3–8 mSv. A preclinical PET/CT system is presented with design features which make it suitable for small animal low-dose imaging. The CT subsystem uses a X-source power that is optimized for small animal imaging. The system design incorporates a spatial beam shaper coupled with a highly sensitive flat-panel detector and very fast acquisition (<10 s) which allows for whole body scans with doses as low as 3 mGy. The mouse total-body PET subsystem uses a detector architecture based on continuous crystals, coupled to SiPM arrays and a readout based in rows and columns. The PET field of view is 150 mm axial and 80 mm transaxial. The high solid-angle coverage of the sample and the use of continuous crystals achieve a sensitivity of 9% (NEMA) that can be leveraged for use of low tracer doses and/or performing rapid scans. The low-dose imaging capabilities of the total-body PET subsystem were tested with NEMA phantoms, in tumor models, a mouse bone metabolism scan and a rat heart dynamic scan. The CT imaging capabilities were tested in mice and in a low contrast phantom. The PET low-dose phantom and animal experiments provide evidence that image quality suitable for preclinical PET studies is achieved. Furthermore, CT image contrast using low dose scan settings was suitable as a reference for PET scans. Total-body mouse PET/CT studies could be completed with total doses of <10 mGy.

## Introduction

Both positron emission tomography (PET) and computed tomography (CT) imaging involves ionizing radiation producing deleterious effects on study animals and constitutes a health risk for researchers. Dose reduction efforts are important in pre-clinical imaging for animal care and study integrity considerations, especially where repeat scans will be performed in longitudinal studies. Further, dose reduction efforts can also help limit the exposure to human system operators. In humans, acute radiation dose effects like skin redness, hair loss, radiation burns, or acute radiation syndrome occur when doses exceed 1 Sv and are delivered at high dose rates. Nevertheless, it is the increase in cancer risk with low but continuous dose exposure that is relevant in radiation exposed workers. Epidemiological studies in humans point to a significant cancer risk increase for doses above 100 mSv (1). The regulation applied in more than ten European countries prescribes the following dose limits: 1 mSv/year for general population and up to 20 mSv/year for professionals. A fundamental edict of dose reduction efforts is that exposures should be kept ALARA (As Lows As Reasonably Achievable).

Efforts to reduce operator and patients' exposure are ongoing (2). Development of total-body PET systems (systems providing simultaneous full body coverage) is receiving increased interest owing not only for the potential to perform whole body screening, capture simultaneous dynamic data for highly quantitative kinetic imaging, but also because the large detector design employed for total body coverage can provide higher sensitivity (3). Total-body PET imaging can provide gains in sensitivity owing to the expanded geometric coverage of the detector design and higher rate of co-incident detections across the axis. Further, total-body PET, in contrast to traditional whole-body PET procedures using axial bed movement which requires image stitching, can provide full sample coverage without increasing total scan time.

CT doses for scans for live mice typically range between 50 and 800 mGy using current scanning technologies (4–7) CT doses for studies of rodent bone metrics and other higher resolution protocols used in for *ex vivo* imaging are commonly >1 Gy (6, 7). CT exposure and dose minimization have been a constant subject of research over the years (8–18).

The scientific literature dealing with PET related doses to small rodents is comparatively scarce. Taschereau et al. used GATE, a Monte Carlo tool, to calculate the PET absorbed doses employing an anatomically realistic 3D mouse model comprising 25 organs and relevant structures (19). The spatial and time distributions of tracer activity were measured through PET scans. It was found that absorbed doses from many preclinical rodent PET protocols can be higher than CT doses depending on activity used and organ. Doses to mice were estimated to be around 100 mGy in the body and as high as 3.7 Gy in the bladder in a typical injection of 7 MBq. Bone marrow and brain doses for that same injection would be around 70 and 100 mGy, respectively. The authors show how doses arising from the PET preclinical imaging procedure can lie well within the range of biological effects in mice and these doses can exceed CT doses by significant margin specially depending on the organ and the activity employed. They recommend emptying the bladder as a standard practice in PET imaging given the large doses.

These doses incurred during preclinical PET/CT scans values are within a range where subclinical effects including DNA breakage and effects on the immune system have been detected (7). On CT imaging doses, Tang et al. report effects of continued or chronic low dose rate on fertility, tumorigenesis and lifespan (20). In addition, induced genetic and epigenetic changes are found as well as cataractogenesis and abnormal neurogenesis in the brain, impairment of neurogenesis and cerebrovascular diseases, resulting in loss of cognitive functions. Tada et al. reported effects in cell proliferation in the dentate gyrus of the brain (21). Klinck et al. found bone architecture changes in skeletally immature mice (14).

Of special relevance on PET/CT Oncology studies is the work of Laforest et al. (9) and Daibes et al. (12) where reduced tumor growth at doses of 60 mGy is reported. In particular Laforest et al. (9) showed tumors receiving higher doses of 110 and 550 mGy would take longer to reach the control tumor sizes. This tumor growth inhibition poses an important argument in favor of protocols minimizing doses.

The exact significance of PET/CT related doses on the animal models under study is complex, but evidence points to influence on the viability of some rodent disease models and bias introduced for the interpretation of the data involving therapeutics.

There are multiple reports covering radiation doses to clinical PET technologists (22–24). In some cases, average annual whole-body doses are reported to be around 3 mSv (22) and in others with high throughput facilities, doses are reported to be around 8 mSv (23). Total exposure varies in relation to the total amount of tracer handled as well as differences in dose preparation and workflows. Demir et al. reported a reduction in doses to 6 mSv after implementing extra radiation shielding (23).

Scientific literature covering doses to pre-clinical PET technologists or researchers is not abundant. However, Prevol et al. report on doses from SPECT imaging with ^111^I (25). In this study, whole-body doses and doses to extremities are comparable to those from the clinic. Interestingly, it is also reported that some of the activities intrinsic to small animal research, like animal dissection, lead to the highest exposures.

Dose per activity coefficients found in clinical studies (22, 23) could suggest a way of estimating doses to pre-clinical PET operators but procedures and isotopes will differ between the clinical and pre-clinical environments, so the result might be unreliable. The fact that lower doses are injected to small rodents than to human patients suggests that doses to pre-clinical operators will be lower. Nevertheless, research labs will operate in less prescriptive workflows than clinical practice and the range of PET isotopes used in research labs will surely be larger than in clinics where [^18^F]FDG will be the dominant isotope. Furthermore, in the preclinical environment, despite that the injected doses will be lower, the vial handled to fill the syringes will contain activity amounts as large as in the clinic. Assuming a hypothetical but realistic high throughput laboratory case of 4 studies per day and 200 annual working days, this leads to 800 studies annually. Taking a 4.1 uSv per study from (22) this would result in approximately 3 mSv annual dose. If the 14 uSv of shallow dose per injection from (24) is used for the same annual work, this would lead to around 11 mSv.

Like in the clinic, strategies to optimize workflows can minimize these dose exposures, but ultimately tracer activity reduction can provide a reliable means to reduce operator exposures. Low-dose PET imaging has the advantage that more animals can be imaged given a certain amount of tracer which can be important when only small amounts are available in some tracer development work.

## Methods

### Material

**Figure 1:**
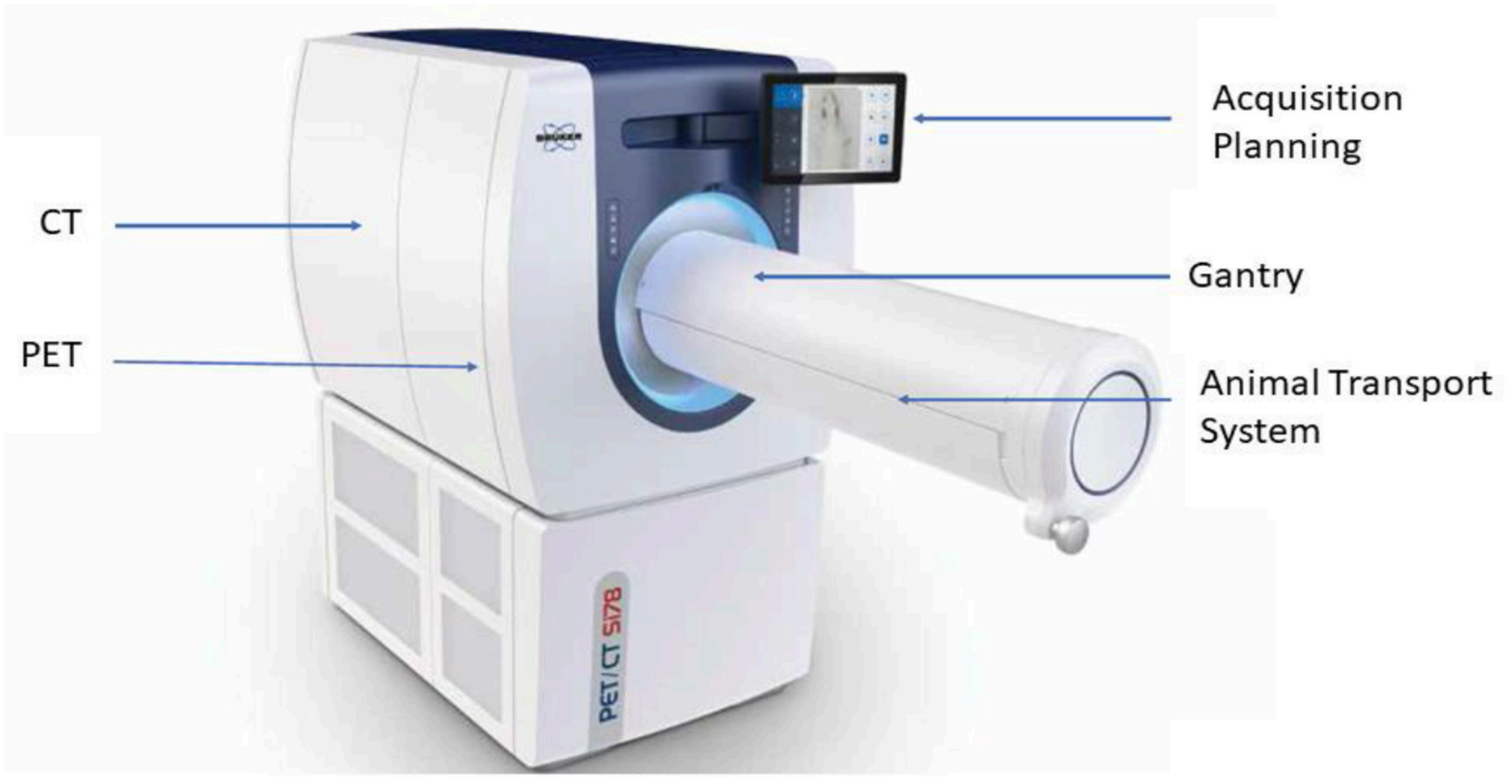
The new PET/CT “Si78”.

**Figure 2:**
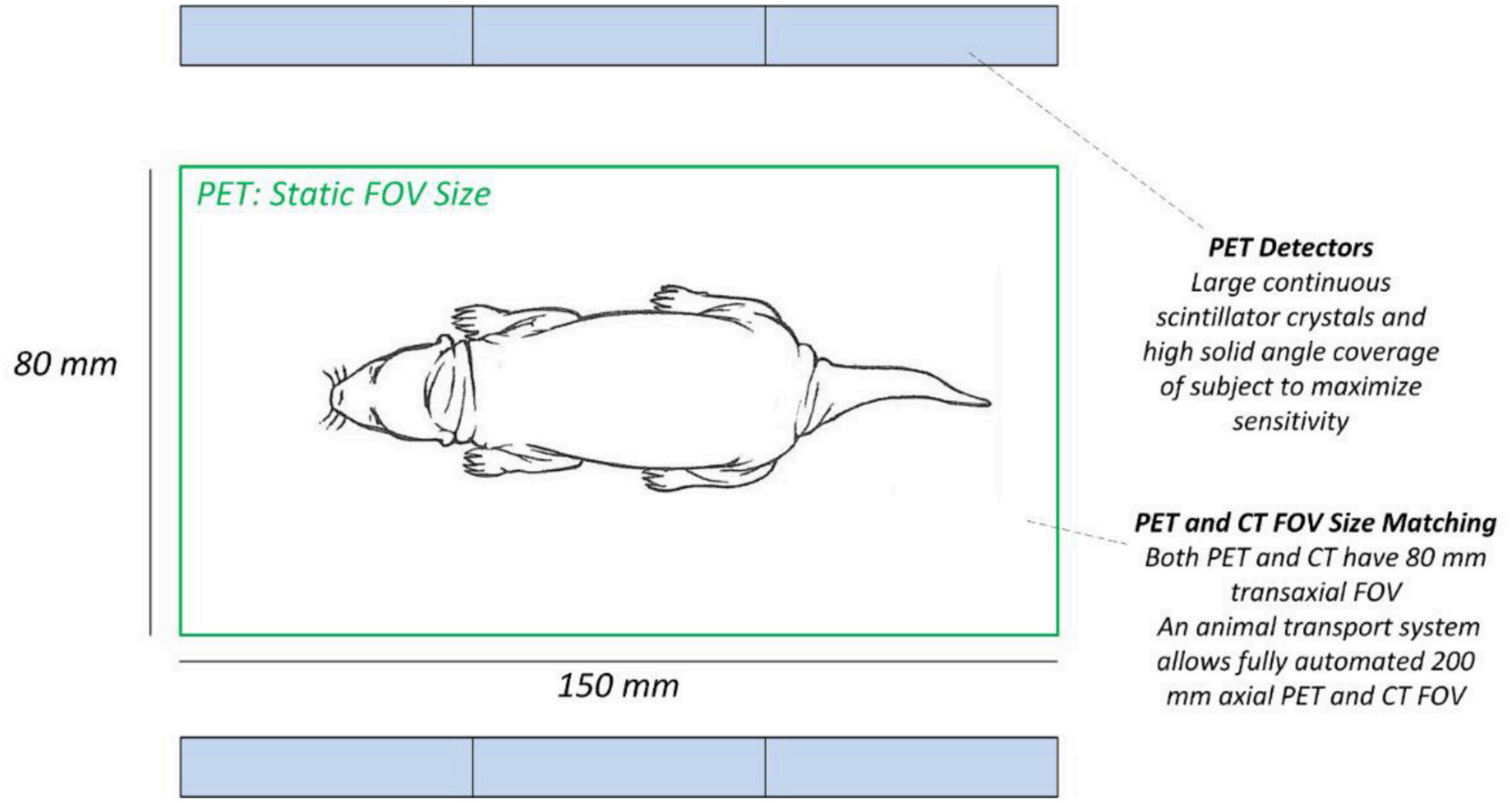
High sensitivity total-body PET detector design. Mouse inside field of view.

An integrated total-body PET/CT system, the Si78, with low-dose capabilities was developed by Bruker Biospin GmbH (Ettlingen, Germany) (see Figure 1). The PET Si78 features a detector architecture based on continuous crystals, coupled to silicon photomultiplier (SiPM) arrays and a readout based in rows and columns (26). A 10 mm thick Lutetium Yttrium Oxyorthosilicate (LYSO) crystal has the shape of a truncated pyramid with a 50 × 50 mm base and a 48 × 48 mm top side. Eight of these units are used to construct an octagonal ring. The transaxial field of view is 80 mm and can be configured with up to three rings for an axial field of view of 150 mm (Figure 2). The sensitivity of the total body PET detector design matches or exceeds that of other current preclinical scanners. Cal-Gonzalez et al. (27) report on a number of existing systems with around 9% sensitivity. The row and column readout approach reduces the amount of SiPM signals that need acquiring from 12 × 12 signals to 12+12. This detector design allows for the characterization of scintillation light pulses which in turn are used to measure the depth of interaction in the crystal, achieving a ≤1.2 mm resolution across the field of view. The high solid angle coverage of the sample and the use of continues crystals achieves a NEMA (28) sensitivity of 9% which in turns allows the minimization of injected activity or scan time. The FPGA based DAQ electronics is capable of simultaneously acquiring a total of 768 detector analog signals achieving a NEMA mouse noise equivalent count rate (NECR) ≥ 500 kcps. The Si78 PET subsystem (detectors and DAQ) has been used in a PET/SPECT/CT platform (Albira Si) as well as in a number of PET/MR platforms. These PET/MR platforms are a PET Insert for simultaneous high field magnetic resonance imaging (MRI) and a PET module present in the PET/MRI 3T system which can also be attached to high-field MRI systems for sequential PET and MRI.

The PET acquisition and reconstructions settings used were all typical of animal work and available on the default software configuration. The energy window employed was 511 ± 153 keV (i. e., 511 keV ± 30%).

Radiation dose in microCT imaging is dependent on three main factors; the x-ray source voltage and current, the distance between the x-ray source and the object (SOD), and the filtration method used. The CT submodule was designed to minimize the radiation dose applied to the animal by using a combination of these factors, and by using additional dose-minimizing components to achieve fast scanning down to <8 s with minimal compromise in image quality. First, an x-ray source with a maximum voltage of 65 kV and power of 50 W is used providing sufficient flux to capture images at a very fast rate. The system is equipped with a flat panel CMOS sensor which has relatively fast frame rates at down to 10 ms. Second, the system is equipped with a set of filters, including a low-dose beam shaper filter, which modify and optimize the x-ray absorption to best suit the animal size that is being scanned.

**Figure 3:**
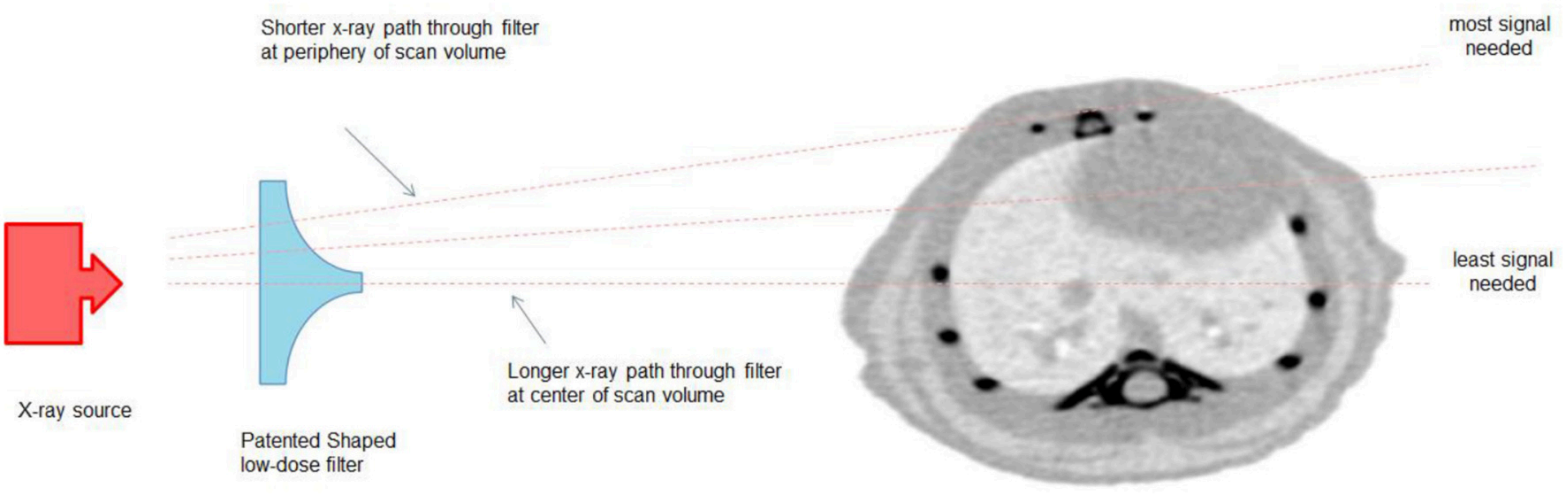
Novel X-ray beam shaper for low-dose imaging.

In 3D CT imaging, there is a redundancy of information collected from the center of the sample. This information tends to be wasted or not used efficiently during the reconstruction process. The low dose beam shaper filter reduces the radiation dose by filtering more x-ray photons from the center of the image which has redundant information and gradually decreases the filtration toward the periphery (see Figure 3). This helps to reduce the radiation dose to the animal by a factor of 2-5 and as low as 3 mGy.

Very low doses can also be achieved using the standard aluminum filters and optimized settings of camera exposure times and source power. The CT subsystem is also used in a standalone CT platform; SkyScan 1278 (Bruker microCT, Kontich Belgium). This CT system has been used for low-dose imaging in studies of skeletal development in mouse pups, in studies of adipose tissue segmentation, and in studies of pulmonary fibrosis. The system has also been used to assess the scan and dose limits where even subclinical radiation dose effects are not incurred (Prof. Vande Velde, KU Leuven, personal communication). Examples of other applications can be found in Xuan et al. (29), Broucke et al. (30) and Covarrubias et al. (31).

### PET Phantom Experiments

Preclinical PET imaging is considered to be the gold standard for quantitative *in vivo* studies. Preclinical PET studies often aim to quantitatively assess new tracers and assess candidate therapeutics through measurement of biomarkers of a range of diseases including cancer and neurological disorders. Tracer activities typically reported in preclinical studies range from 5 to 40 MBq.

Initial phantom experiments were performed to assess the data integrity, relevant to common preclinical studies, when imaging low tracer activities using a preclinical total-body PET/CT system.

To assess ability of the PET system to accurately distinguish small activity difference in low-dose imaging a NEMA mouse size scatter phantom was loaded with 925 kBq [^18^F]FDG and imaged over 60 min.

The NEMA Image Quality Phantom was used to assess the uniformity, recovery coefficients for the different rod sizes as well as the spill over ratio for water and air. The phantom was loaded with 1 MBq [^18^F]FDG and after 10 min, a static acquisition of 20 min was performed. The IQ phantom acquisition was reconstructed with the maximum likelihood expectation maximization (MLEM) algorithm using 25 iterations and a voxel size of 0.5 mm. In addition, a point spread function iterative deconvolution based Partial Volume Correction (PVC) was employed (32).

To assess the spatial resolution in low dose imaging employing realistic laboratory conditions a micro-Derenzo type phantom was loaded with 3 MBq and acquired for 20 min. The reconstruction settings employed were also equivalent to those typically employed in animal experiments: MLEM algorithm, 30 iterations, voxel size of 0.25 mm and PVC activated.

### PET *in vivo* Experiments

Preclinical PET is used widely in studies of tumor biology and tumor progression. Such studies often aim to measure quantitative changes in tumor biomarkers. Initially, the low dose imaging capabilities of the total-body PET subsystem were tested as part of an ongoing breast cancer model experiment in mice. Here only one mouse was imaged as a preliminary study to investigate the viability of this technique. As a further low dose imaging test, 2 mice were imaged as part of an ongoing fibrosarcoma experiment. In both experiments, data was assessed for contrast, resolution, and quantitative integrity critical for small animal tumor studies. On the breast cancer model, an immunodeficient BALB/c nude female mouse was imaged 10 days after MCF-7 breast cancer cells were grafted being the animal 7 weeks old. The mouse drank water supplemented with β-estradiol. On the fibrosarcoma experiment, two immunodeficient BALB/c nude female mice were imaged 10 days after SK-N-AS fibrosarcoma tumor cells were grafted being the animals 7 weeks old. In both experiments, acquisitions were carried out 70 min after the injection of 1 MBq of [^18^F]FDG and lasted 60 min. The whole of the list-mode file was rebinned in 10 min frames and an image was reconstructed out of each frame. The PET image reconstruction settings were: MLEM algorithm, 24 iterations and a 0.5 mm voxel size.

The [^18^F]FDG dose/MBq coefficients obtained by Taschereau and Chatziioannou (19) for the body, brain and bone are 14, 13, and 5 mGy/MBq, respectively. For the radiosensitive organs like testes and thyroid, these coefficients were 16 and 14, respectively. The bladder was the organ with the largest dose value with over 500 mGy/MBq. The dose/MBq coefficient for the body was used to derive the dose due to the PET procedure in this experiment.

Researchers are commonly employing multi-animal beds and simultaneous imaging to increase preclinical PET throughput. Three C57BL/6 female mice were imaged simultaneously using a low-dose protocol using the [^18^F]NaF tracer. Animals received 2 MBq [^18^F]NaF and were scanned 40 min post injection. List-mode data was reconstructed at variable effective scan times to determine the minimum acceptable scan time. The list-mode file was divided in 1 min frames and reconstructed at low resolution to check whether the animals had remained still. Twenty minutes into the acquisition the animals had remained still for 18 min whereas in the rest of the acquisition there had been some motion of tails, spine and bladder causing significant blurring. Uptake time at the start of the selected list-mode frame had been 1 h and each of the animals had an activity of 1.3 MBq in the mid-point of the frame. The selected 18 min were reconstructed with using MLEM, 24 iterations and a voxel of 0.25 mm. PVC (32) was applied and the final image was filtered using a Median 3D algorithm with a 1.0 mm isotropic kernel.

In the case of fluoride ion, the dose/MBq coefficients obtained by Taschereau and Chatziioannou(19) for the body, brain and bone are 10, 13, and 76 mGy/MBq, respectively. Testes and thyroid were 9 and 5, respectively with the bladder getting over 300 mGy/MBq. The dose/MBq coefficient for the body was used to derive the dose due to the PET procedure in this experiment.

Dynamic PET imaging is a tool for testing and validating tracer kinetic modeling. Tracer kinetic modeling is a method for extracting transfer rate constants, volumes of distribution or binding potentials from the radiotracer to the target, by describing the mechanism of transport and biochemical reactions of the tracer in tissue. Blood samples are often taken from the animal to obtain the so-called input function. This is compared to the PET image to obtain the dynamics of the radiotracer concentration in local tissue over time.

A further PET experiment using a control Wistar Kyoto (WKY) was also carried out to investigate the applicability of low dose imaging in [^18^F]FDG dynamic cardiac imaging. The imaging protocol was similar to already published work in mice and more recently in rats (33–35). The rat was fasted for 5–6 h with free access to water prior to [^18^F]FDG imaging. A 60 min dynamic PET data acquisition was initiated a few seconds before the slow (over 10-20 s) administration of 2.7 MBq [18F]FDG via a tail-vein catheter. Blood samples were also collected from tail-vein right of the rat after PET/CT scan in a volume of 0.36 ml, which were then weighed and radioactivity measured using a Hidex Automatic Gamma Counter. The decay corrected blood sample measurement at 56 min was used for validation of model corrected blood input function at the same time point, as described (34, 35). The list-mode data was sorted into 23 time bins and reconstructed with attenuation correction using MLEM algorithm with 6 iterations and 0.75 mm isotropic voxel resolution. Region of interest (ROI) analysis and computation of myocardial FDG uptake rate, Ki, was performed as described (33–35). Data representing [^18^F]FDG concentrations in the myocardium and left ventricle were taken from two ROIs in a single frame of the 4-D dynamic PET image. The slices from the last time frame of the image were manually parsed until the left ventricle was clearly visible. Once a slice in the midsection of the heart was identified, the two ROIs were manually drawn as continuous lines using a series of connected points. Time activity curves (TAC) for each of the two ROIs were generated by averaging the image data of a given ROI at each time frame. Due to the intrinsically limited spatial resolution offered by PET as an imaging technique and the small size of rat heart, partial volume (PV) averaging can become a significant problem resulting in spill over contamination (SP) from the blood to the tissue at early and vice-versa at late time points. Using the formalism developed in Zhong et al. (33), Zhong and Kundu, (34), Li et al. (35), and Li and Kundu. (36), a 3-compartment kinetic model that can simultaneously correct for SP and PV effects for both the blood pool and myocardium, generate a model corrected blood input function (MCIF) and compute kinetic rate parameters (K1-k3), was used to compute rate of myocardial FDG uptake, Ki (35). The above analysis was performed using the MATLAB_r2018a (Mathworks Inc., Natick MA) computing environment.

Given the absence of appropriate dose/MBq coefficients for rats, doses were not calculated in this experiment.

### CT Dose Calculations and Measurements

The Bruker's software CTion 1.0 was used for the calculations. The calculation of X-ray dose in CTion is based on the simulations of X-ray photon energy spectra and dose by the program SpekCalc (37–39). X-rays are assumed to be emitted from an X-ray source using a tungsten target, as used on the Si78. CTion calculates dose rates in mGy/min in air, for a rat and for a mouse once the user inputs these values: current, voltage, distance source to sample and type of filtering.

In order to assess the accuracy of the calculations, dose measurements were also carried out with a MOSFET type dosimeter. In particular, the Unfors Patient Skin Dosimeter was used for the measurements. This features two MOSFET (Metal Oxide Semiconductor Field Effect Transistor) detector heads, one is in air to measure air dose, the other was shielded by a white PMMA tube with overall thickness about 25 mm to estimate depth-corrected dose in a mouse.

### CT Phantom Experiments

In order to assess tissue contrast obtained with CT settings which would suffice for a PET scan anatomical reference, a low contrast CT phantom (QRM) was scanned at a range of settings. Dose estimates were derived from simulations by CTion.

Furthermore, to assess the general CT image quality and specifically uniformity in low dose imaging, a phantom consisting of a water tube was scanned at two different resolutions, 200 and 100 micron resolution. The CT settings were such to mimic the typical conditions in a preclinical PET/CT experiment.

### CT *in vivo* Imaging of a Wild Type Mouse

A first experiment was designed to achieve the best image quality possible while minimizing the dose. An adult wild type mouse was scanned using a Low Dose 1 mm Al filter at 39 kVp and with varying current and acquisition times to progressively lower the dose and assess the resulting image quality. Keeping the current at 199 μA, two scans were performed for 149 and 84 s, respectively. Then, lowering the current to 69 μA, two scans were performed for 91 and 44 s, respectively. The exposure time was kept at 300 ms. The data was reconstructed using the filtered back projection (FBP) algorithm and standard reconstruction parameters.

In a second experiment, an adult wild type mouse was scanned minimizing both acquisition time and dose. The settings were 54 kVp, 900 μA and 7 s of total scan time resulting in a 200 um pixel resolution image. The option to scan using “continuous mode” instead of “step and shoot” was used to achieve the fast scan times. The data was reconstructed using the FBP algorithm and standard reconstruction parameters.

## Results

### PET Phantom Experiments

**Figure 4:**
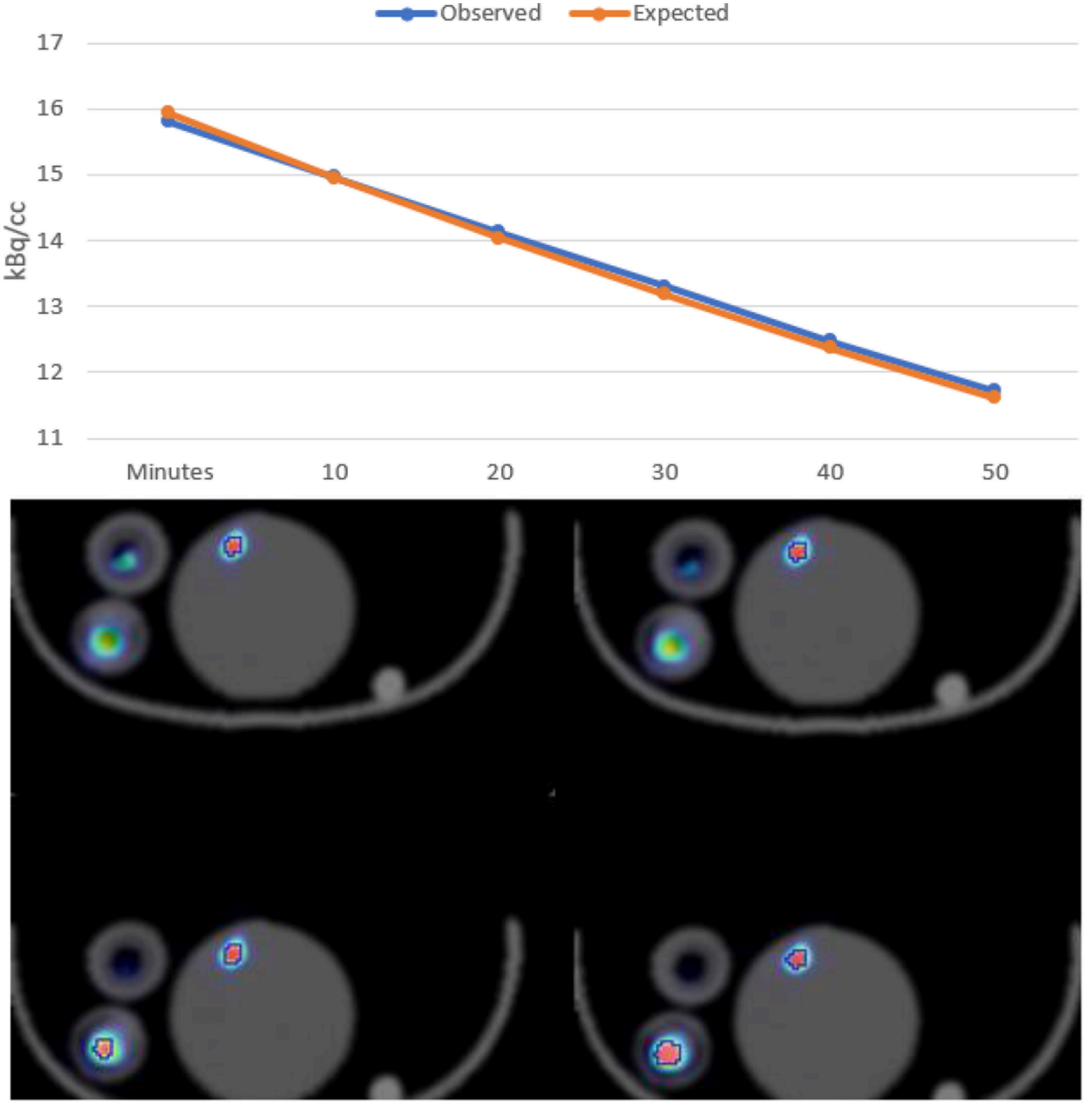
Mouse NECR Phantom [^18^F]FDG starting 925 kBq signal quantified over 60-min decay.

Quantitative assessment of the low activity during the decay period in the mouse NECR phantom revealed that subtle difference in activities could be distinguished and that measured values were consistent with expected results (see Figure 4).

**Figure 5:**
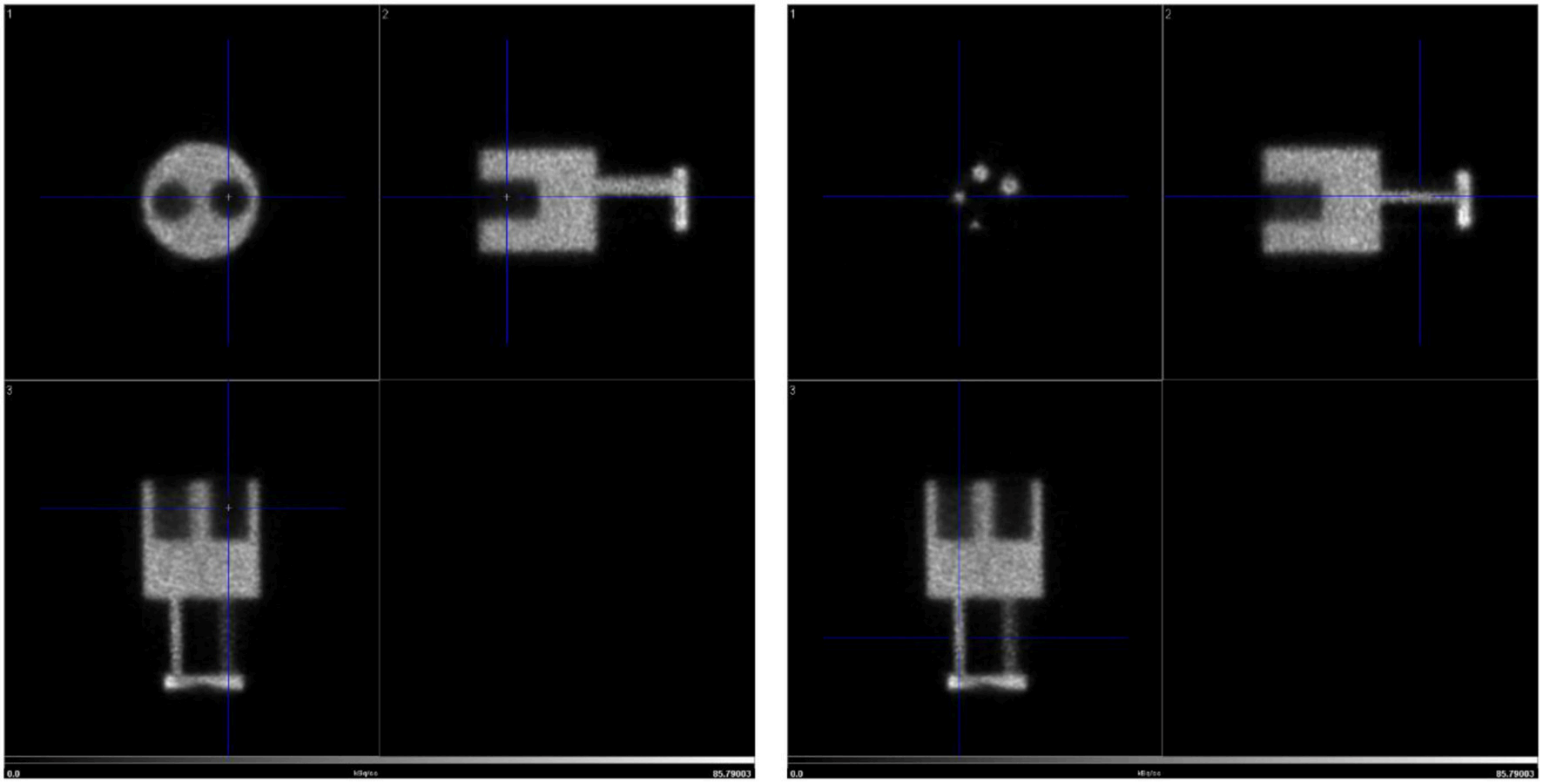
IQ Phantom images acquired over 20 min with <1 MBq.

Figure 5 shows the IQ Phantom with a transversal slice through the SOR region of the phantom and another one through the capillaries. Tables 1A–C show the numerical analysis of the IQ phantom. NEMA Image Quality with PVC (Partial Volume Correction) shows recovery coefficients close to 1.0 for all fillable rod sizes but the 1 mm size rod (Table 1B). Nevertheless, resulting standard deviations are relatively high.

Table 1NEMA IQ results.

**Table:** 

**A. Uniformity**				
	**Mean**	**Maximum**	**Minimum**	**Std (%)**
PVC OFF	49.2 kBq/ml	71.7 kBq/ml	32.2 kBq/ml	9.6
PVC ON	49.2 kBq/ml	94.9 kBq/ml	14.2 kBq/ml	19.6

**Table:** 

**B. Recovery Coefficients. Standard Deviation in % Within Brackets**					
	**1 mm**	**2 mm**	**3 mm**	**4 mm**	**5 mm**
PVC OFF	0.04 (28.5)	0.68 (17.3)	0.88 (13.3)	1.00 (15.5)	0.96 (15.5)
PVC ON	0.04 (52.8)	0.92 (30.6)	1.02 (26.3)	1.13 (25.6)	1.03 (30.7)

**Table:** 

**C. Spill Over Ratio. Standard Deviation in % Within Brackets**		
	**Air**	**Water**
PVC OFF	0.17 (35.0)	0.25 (23.0)
PVC ON	0.16 (42.9)	0.25 (35.1)

PVC has therefore a positive effect bringing the 2, 3, and 5 mm rods recovery coefficients closer to 1.0, especially the 2 mm rod. However, an increase in image noise is also observed when PVC is applied. This is especially evident from the uniformity results (Table 1A), where ideally there is no influence of partial volume.

**Figure 6:**
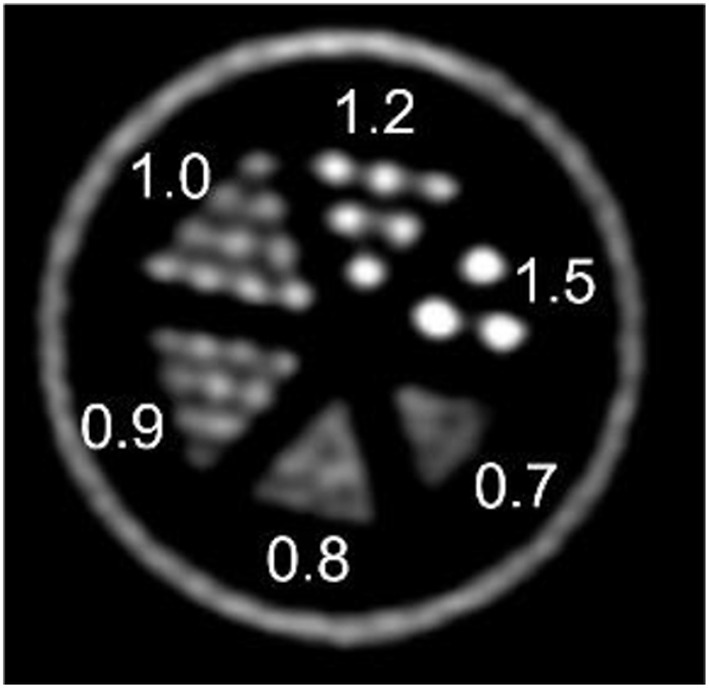
micro-Derenzo phantom imaged for 20 min with 3 MBq of [^18^F]-FDG. Capillary sizes in mm are shown in the image.

Figure 6 shows the resulting image of the micro-Derenzo type phantom experiment. Capillaries in the diameter range of 0.9–1.2 mm are well resolved highlighting the possibility of submillimetric resolutions even in low dose animal experiments.

### PET *in vivo* Experiments

**Figure 7:**
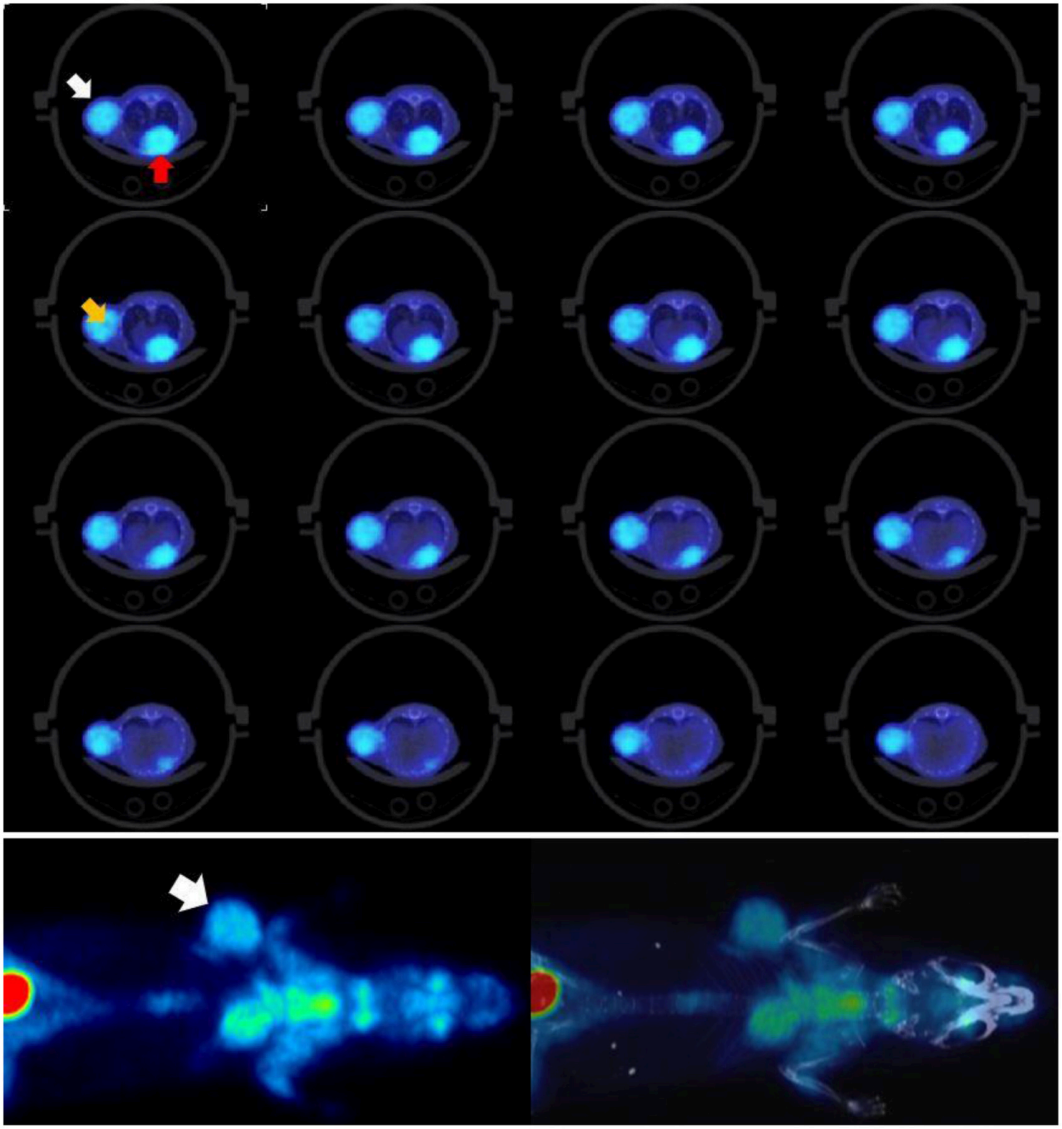
Mouse 1 of fibrosarcoma tumor model. **Top:** Tumor is marked by white arrow and heart by red arrow. Contrast and resolution are sufficient to resolve heterogenous tumor uptake, see orange arrow. **Bottom:** Low-dose total-body [^18^F]FDG/PET with clearly defined tumor uptake and contrast. Tumor uptake measurements (13% ID/mL) obtained fall within typical ranges for this model. Results for 10-minute and 60-minute scans are consistent, indicating that low-dose imaging even for 10 minutes provides sufficient counts for reliable data and quantifications.

To evaluate the system for use in low-dose preclinical PET imaging one mouse with a breast tumor and two mice with a fibrosarcoma tumor model were employed. When the %ID/mL calculated from the last frame image was compared with the rest of frames, and also with the whole list-mode file image, this showed a good agreement. The image of this last frame also showed adequate quality tumor uptake was 11 %ID/mL for the breast model and around 13 %ID/mL for the fibrosarcoma model. Figure 7 shows mouse 1 of the fibrosarcoma experiment.

By using the Dose/MBq conversion coefficient explained in Taschereau and Chatziioannou (19) the whole-body dose due to the PET procedure was estimated to be 14 mGy but based on the results obtained here this could likely be lowered to at least 7 mGy. The activity in the midpoint of the last frame was 452 kBq and this suggests that injected doses can at least be halved and should not affect the experiment results significantly.

These results suggest the possibility of using low dose injection in this particular tumor model. Nevertheless, more animals would have to be imaged to make the result statistically significant. Also, it is still to be seen the applicability of low dose imaging for both other tumor models as well as other tracers.

**Figure 8:**
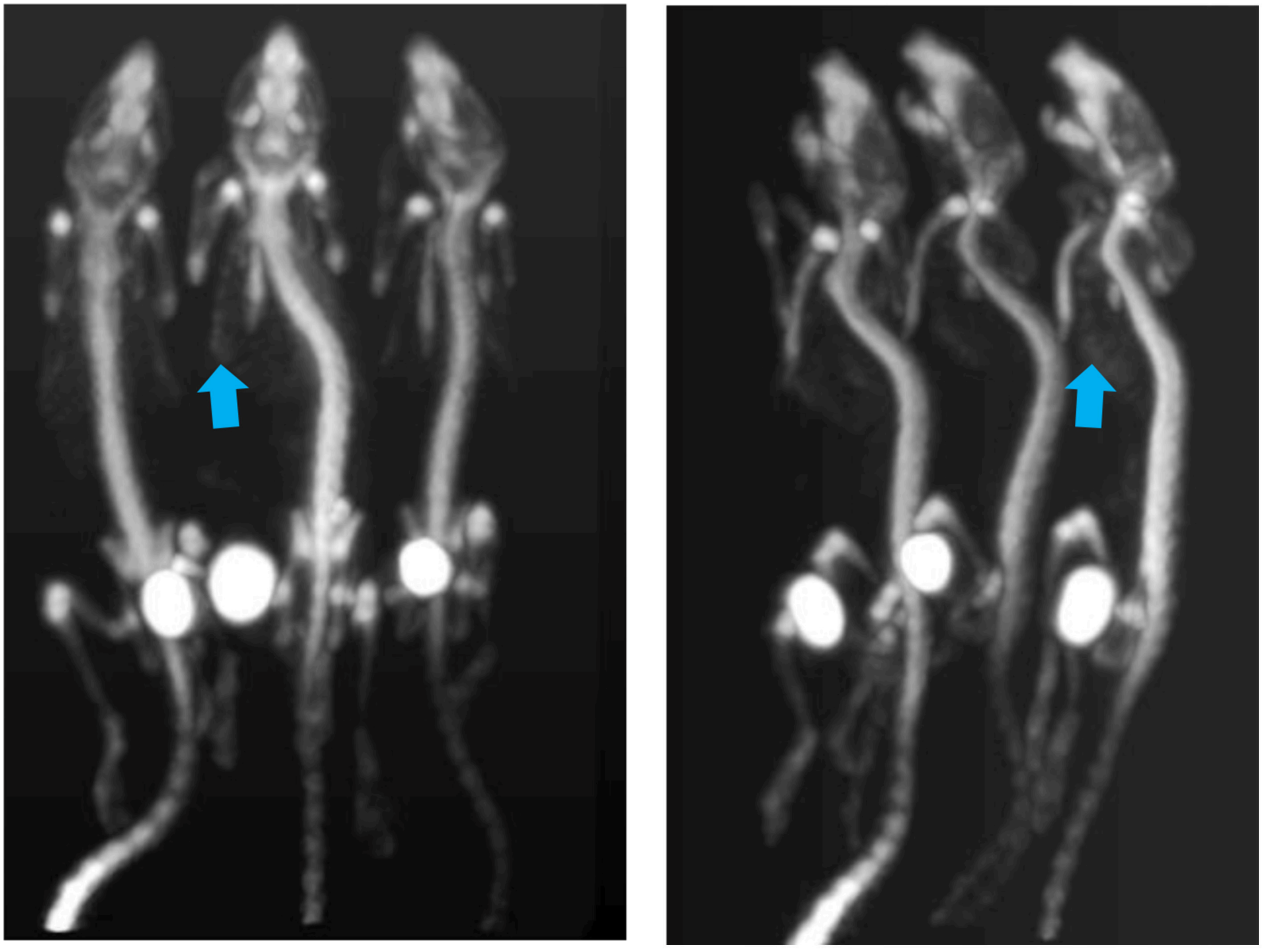
PET MIP images showing bone tracer Na[^18^F]. Arrows point to visible ribs as an indication of good resolution. Tracer uptake time was 1 h and scan time 18 min. Each of the animals had been injected with 2 MBq.

Figure 8 shows the resulting MIP image for the total body of all 3 mice.

Using the provided dose activity conversion coefficient of 10 mGy/MBq (19) for fluoride ion therefore it can be estimated that each of the animals received a dose of 20 mGy just from their injection. The total dose to each of the animals in this multiple animal experiment will nevertheless be higher given the external irradiation from the neighboring animals. The images show that sufficiently good resolution is achieved in low dose experiments in acquisitions lasting <20 min.

**Figure 9:**
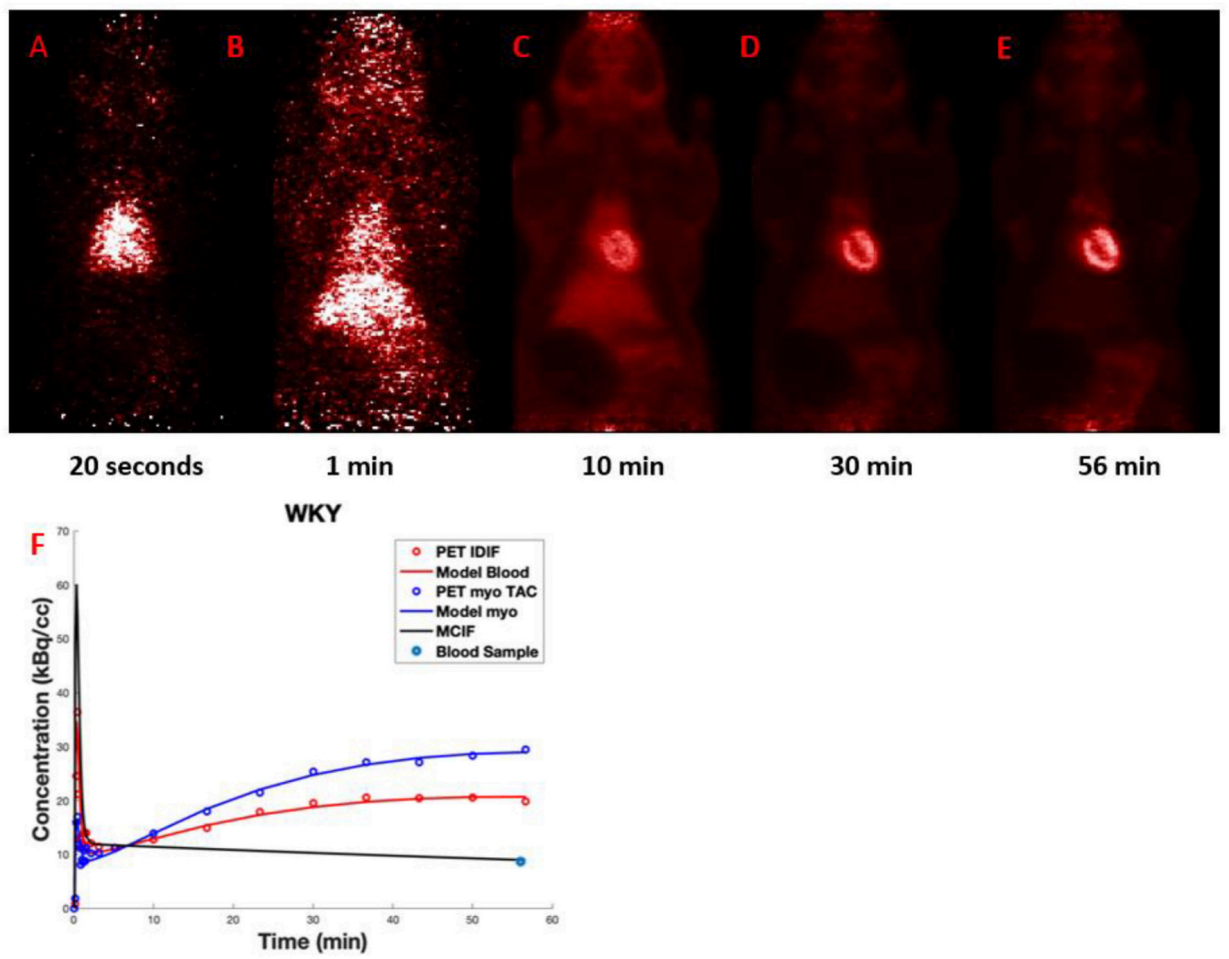
**(A–E)** Dynamic FDG PET images: Representative images at 20 s, 1, 10, 30, and 56 min post FDG administration. **(F)** Time activity curves: Representative time activity curves for LV blood pool and myocardium, model fits, and model corrected blood input function (MCIF). The MCIF at 56 min agrees with a blood sample (8.67 kBq/cc) at the same time point.

Figures 9A–E shows representative dynamic [^18^F]FDG PET coronal images for the rat heart *in vivo* experiment at an early time frame (20 s), and at 1, 10, 30, and 56 min post [^18^F]FDG injection. The latest time frame most clearly showed the myocardium, see Figure 9E, since most [^18^F]FDG had been taken up from the blood by that time point. Figure 9F shows representative time activity curves for LV blood pool and myocardium, model fits, and MCIF. The MCIF at 56 min agrees with a blood sample (8.67 kBq/cc) at the same time point.

This experiment has shown the feasibility of low dose imaging also in the context of dynamic [18F]FDG PET with rats.

### CT Doses Calculation and Measurement

For the setting of 40 kV, 500 μA, and 1 mm Al filtration, the absorbed X-ray dose calculated by CTion, in air and averaged over a mouse of 25 mm diameter, were 47.95 and 26.07 mGy/min respectively. With these same settings the doses measured by the MOSFET Patient Skin Dosimeter, in air and shielded by about 25 mm thickness of PMMA, were 44.5 and 20.0 mGy/min respectively. Thus, the deviation between the doses calculated by emission simulation and measured by MOSFET detector, were 8 and 30% for the dose in air and the dose depth corrected for mouse, respectively. Generally, it was observed that for lower voltages and filtering the MOSFET-measured values were lower than the simulated values. The contrary was observed with higher voltages. The agreement for 50 kV was within 10% on both air and mouse doses. This could be explained by the decrease of detection efficiency of the MOSFET type dosimeter detectors employed, with decreasing photon energies below about 30 keV X-ray photon energies in CT imaging, even with aluminum filtration, extend down to 10–15 keV. The CT settings in typical PET/CT experiment use step and shoot and a 200 μA current so based on the results above, the dose to mouse scales linearly with current and therefore can be estimated to 10 mGy/min. Air doses would be around 19 mGy/min.

### CT Phantom Experiments

**Figure 10:**
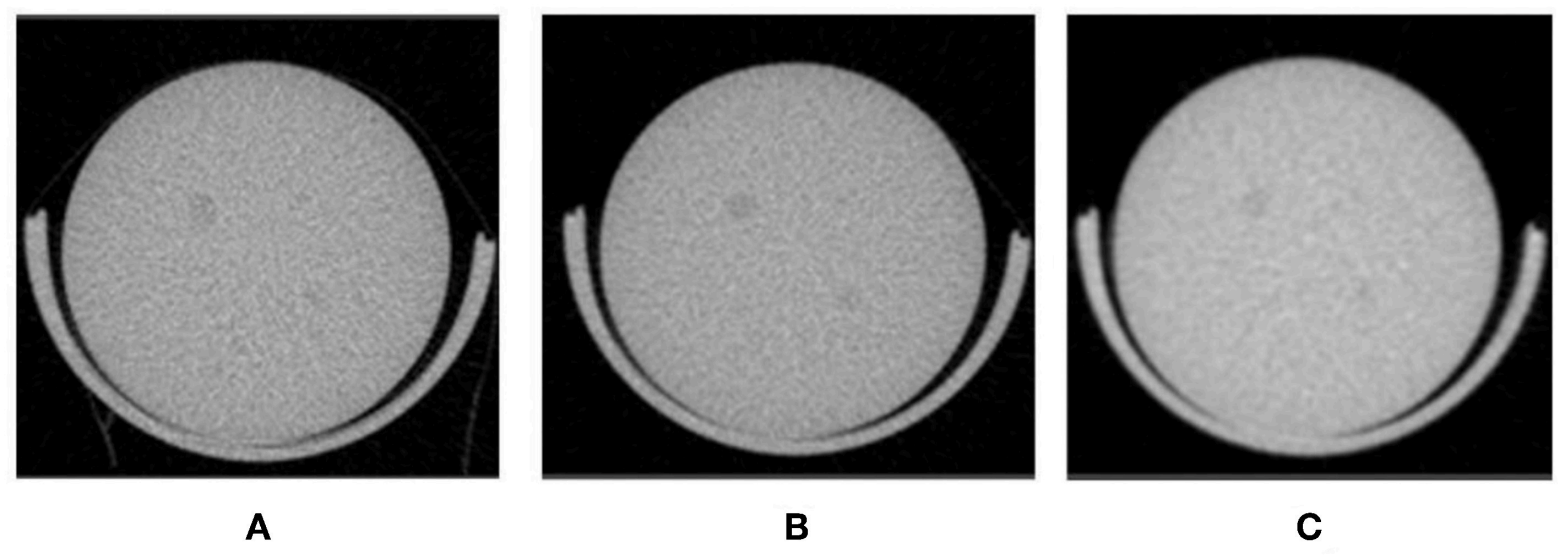
Contrast phantom to evaluate contrast at low-doses **(A)** Scan time: 140 s and dose 12.5 mGy **(B)** 57 s and 9.9 mGy **(C)** 7 s and 5.8 mGy.

Figure 10 shows how imaging of a low contrast phantom reveals sufficient contrast to detect 2.5 mm rods. Rods were imaged from seconds to minutes and using variable (time, Al filter, current, and voltage). Contrast for phantom imaging can be enhanced further (not shown) with modified settings, with modest increases in dose.

**Figure 11:**
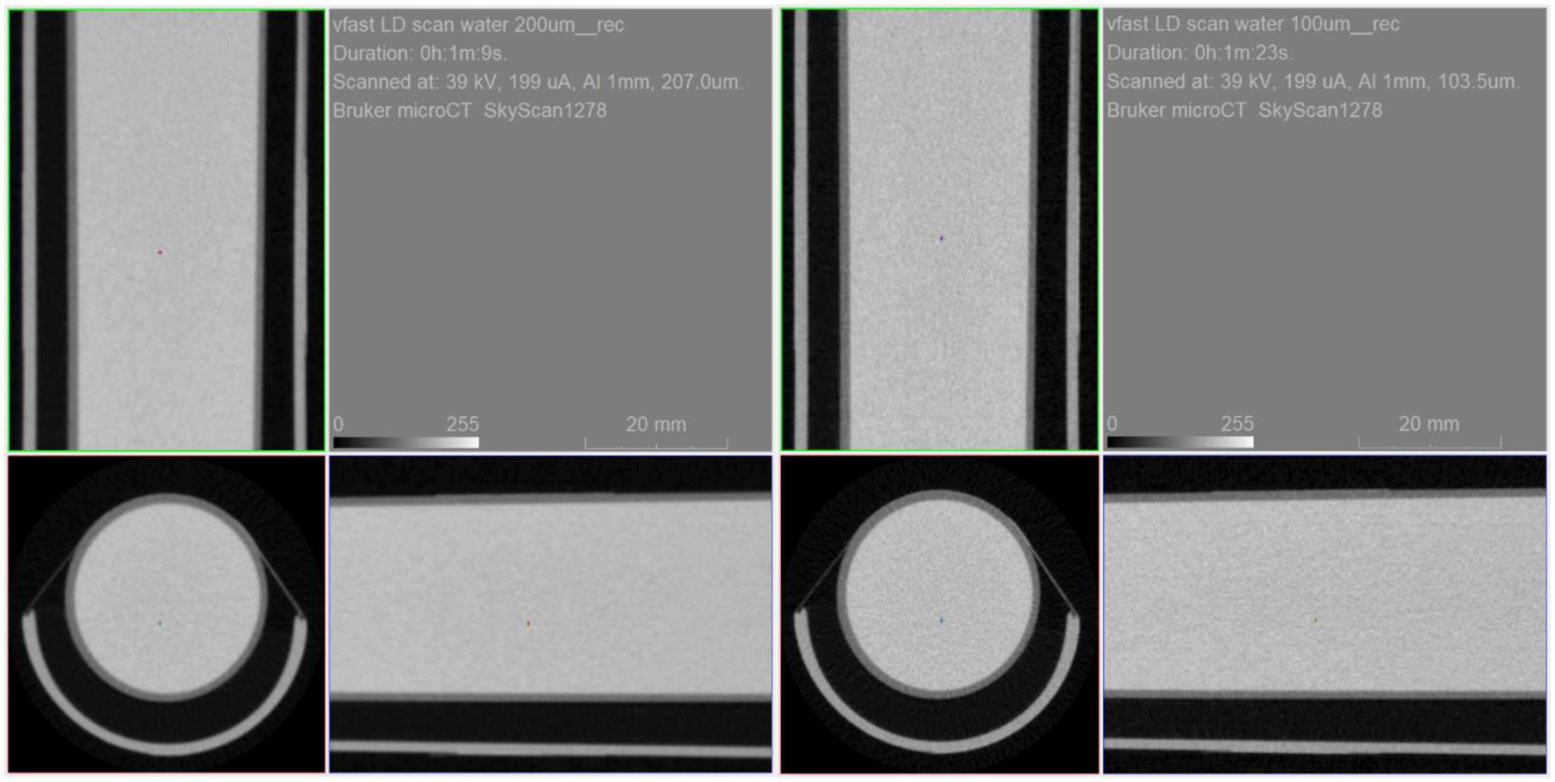
Water tube phantom scanned with 200 um resolution (left) and 100 um (resolution) in low dose setting mode.

The water tube phantom experiment was carried out using the step and shoot mode and employed the low dose settings: 40 kV, 200 μA, 1 mm Al filter and 300 ms exposure time. Those are estimated to be in the 10–15 mGy range. Each acquisition took just over 1 min of scan time. Figure 11 shows the resulting uniform artifact free images.

### CT *in vivo* Imaging of a Wild Type Mice

**Figure 12:**
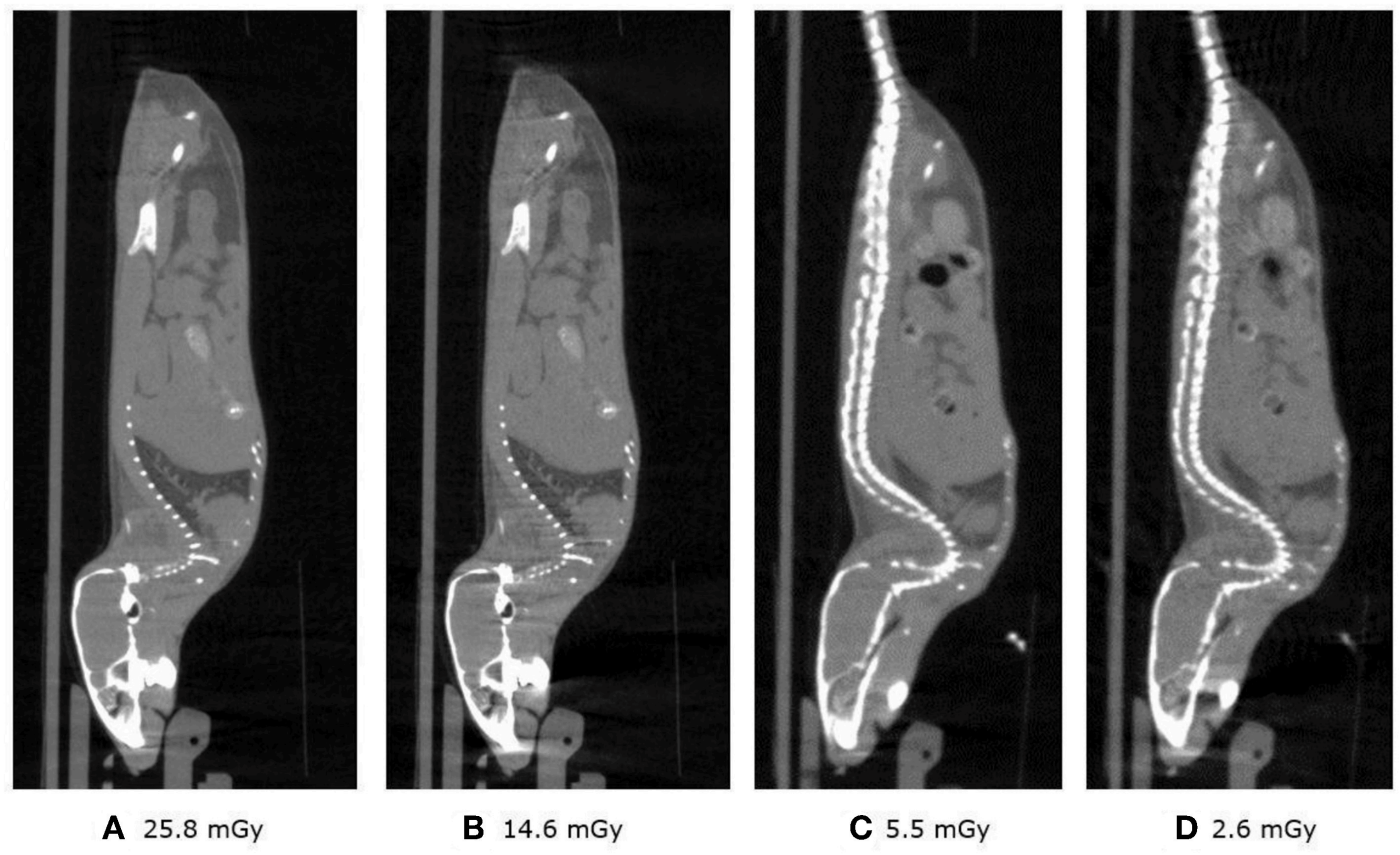
**(A–D)** Above show the 4 sagittal views with current and acquisition time settings such that approximately the dose is halved starting at 25.8 and finishing at 2.6 mGy.

**Figure 13:**
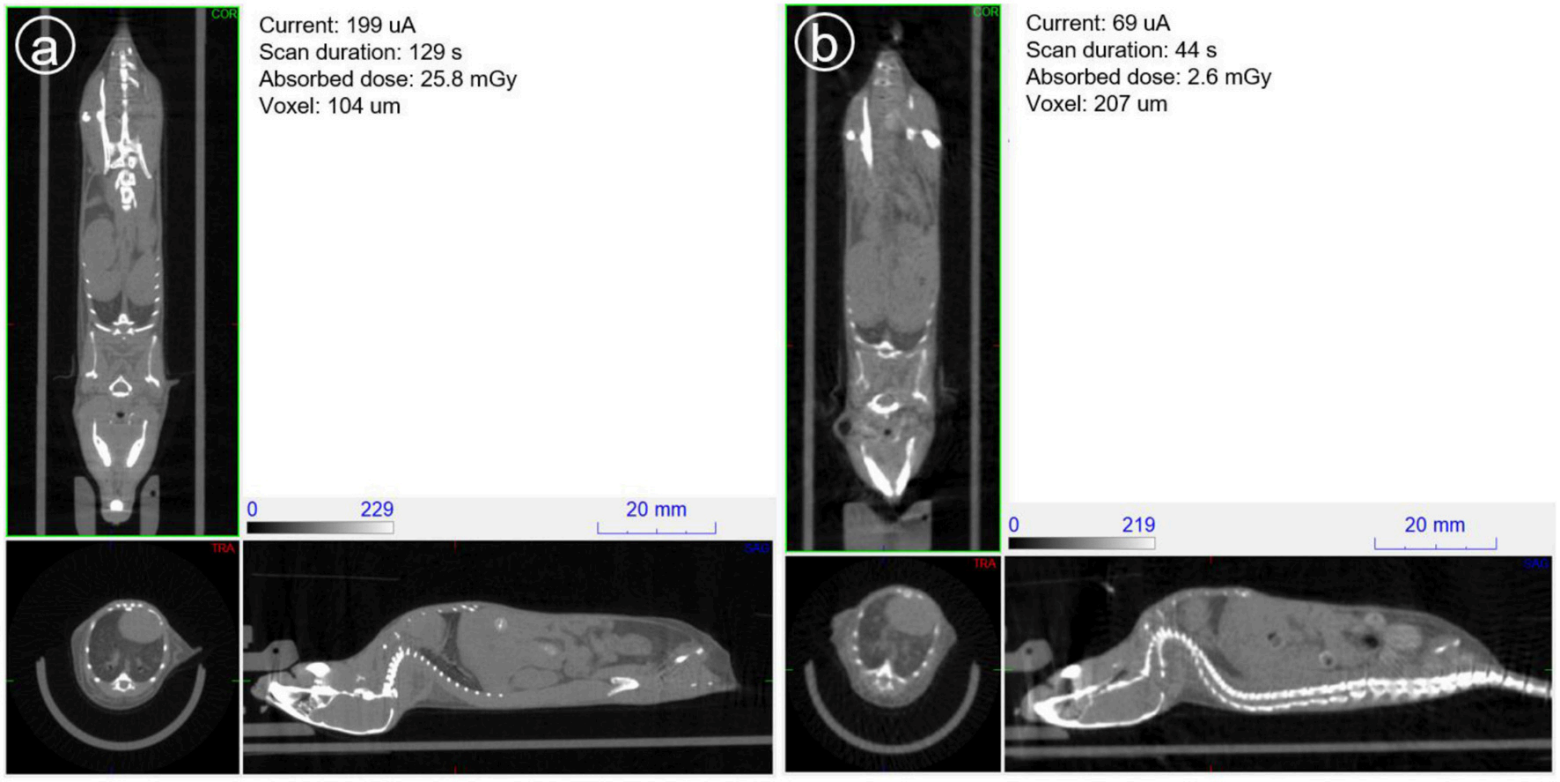
Mouse scanned at 39 kV and aluminum filtering of 1 mm. **(a)** On the left with typical imaging settings and **(b)** on the right, current and scan duration optimized to minimize dose.

Figure 12 shows the sagittal view of the mouse imaged with 4 different settings in which dose is reduced from a starting point of 26, then to 15, 16 and finally 3 mGy approximately. It can be seen how skeletal and tissue contrast decreases as dose goes down but stays even in the 3 mGy case sufficiently good for anatomical reference in small animal PET scans. Figure 13 shows the 26 and 3 mGy cases next to each other including all three coronal, sagittal and transversal views.

This experiment shows how with 40 kV and 1 mm Al filtering, low dose images of enough quality can be obtained in less than a minute by using relatively long exposure times (e.g., 300 ms) and low current.

**Figure 14:**
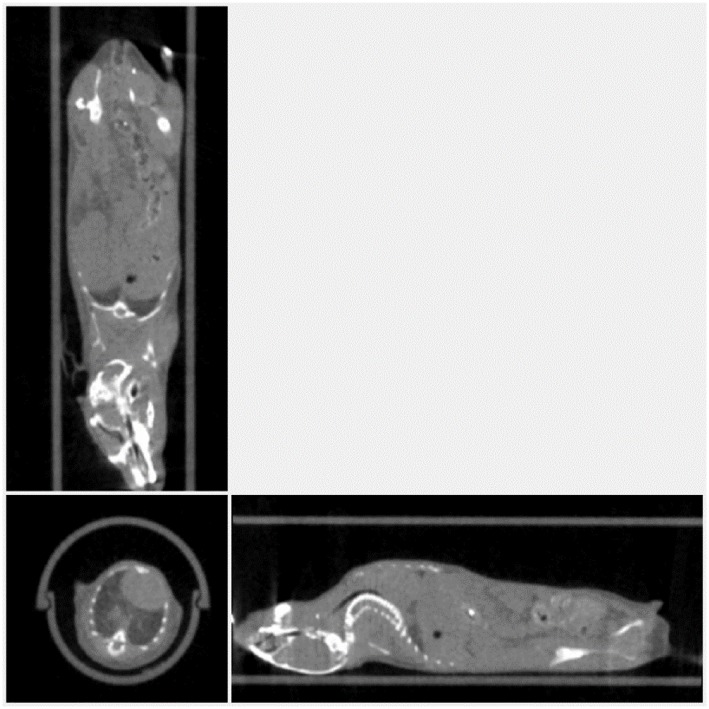
Mouse imaged with 54 kVp, 900 μA, and an acquisition time of 7 s and dose <10 mGy. The image voxel is 200 um.

Figure 14 shows the result of the experiment to minimize not only dose but acquisition time. The image shown is the result of a 7 s scan time and the dose is calculated to be less 10 mGy. Again, dose reduction settings provide sufficiently good contrast for skeletal structure, as well as lower contrast soft tissue organs.

## Discussion and Conclusions

Low-dose imaging protocols in phantoms and animals were used in the Si78, a new preclinical total-body PET/CT system.

CT imaging serves as an anatomical reference for functional PET detections using integrated PET/CT systems. Anatomical detections are critical for localizing PET signal and providing a backdrop for accurately defining regions of interest for analysis. The Si78 CT subcomponent was assessed for contrast and image quality using low-dose protocols. Low-dose imaging provided sufficient contrast to resolve tissue contrast to serve as an anatomical reference for small animal PET scans.

Given that low-dose PET imaging can be performed with doses <4 times lower relative to standard protocols, system operators can benefit from reduced accumulative annual dose exposures simply through reduced dose handling. This could be particularly critical in core facilities where dedicated personnel perform much of the scanning and tracer handling for an institution. The new instrument allows for total-body mouse PET/CT studies with doses of <10 mGy for each the PET and CT procedures, minimizing animal dose exposures.

Low-dose PET imaging was shown to provide not only qualitative features valued in functional imaging studies, but contrast, resolution and quantitative features typical of standard protocols. Low-dose PET imaging in a preclinical tumor model provided suitable resolution and quantitative measurements. Often, studies aim to identify unique features of tumor microenvironment that are believed to be critical to the progression of many tumors. Regional changes in stromal tissues, metabolics, and receptor expression are believed to be indicative of such processes. Significantly, image resolution was sufficient to distinguish intratumor metabolic heterogeneities, indicating that low-dose imaging in this system will be suitable for interrogating tumor biology critical for tumor progressions. Given that preclinical PET imaging is often used to make quantitative measurements of various molecular and cellular processes, low-dose PET protocols should provide linear quantitative detections. Linear dose decay measurements at <1 MBq in a mouse phantom demonstrated that even subtle concentration difference could be distinguished, providing evidence that quantitative measurements at low doses are not compromised.

Further, low-dose multi-animal imaging using [^18^F]NaF demonstrated that fine details of bone metabolism could be resolved, providing further evidence that low-dose imaging can provide total-body contrast sufficient for resolving details across all anatomies covered in a total-body scan. One of the key benefits for total-body PET imaging is the ability to collect total-body dynamic data appropriate for highly quantitative kinetic modeling that surpass traditional semiquantitative SUV measurements. Total-body high resolution demonstrated here would indicate that imaging at both local and distant anatomies could be employed for total-body kinetic modeling studies.

The rat heart dynamic PET experiment has also shown the feasibility of low dose imaging for such an application.

Low-dose high-sensitivity PET imaging provides several cost-saving and performance benefits beyond simple reduction in exposures to ionizing radiation. Low-dose PET imaging can be particularly valuable in studies of new candidate tracers. Often, development of candidate tracers can be costly where tracer chemistries and ligand development may be made in an iterative process. Low-dose PET imaging can be useful because it allows for testing of very low-volume and low-activity tracers and tracer performance is validated and production is scaled. In fact, even for routine imaging using established tracers, low-dose imaging allows researchers to reduce costs related to tracers. Low-dose (high sensitivity imaging) can also be useful for sensitive cell tracking studies. Further, high-sensitivity imaging can alternatively be used to reduce total exposure times when standard tracer activities are used, resulting in gains in throughput.

Low-dose CT imaging in mice demonstrated that suitable tissue contrast and resolution were achieved. Using a combination of dose minimizing energies and hardware, including a beam sharping filter, supply suitable images/data. Anatomical scanning could be achieved with total doses that were well below even limits of subclinical effects, providing absolute confidence for researchers that results/conclusions will not be compromised by scanning protocols and as of yet undetermined effects of subclinical effects of X-ray exposures on sensitive disease models. Further, the CT scanning and reconstruction can be completed at high-throughput (<10 s ) suitable for screening multiple cohorts of animals commonly used in studies of new therapeutics. Significantly, the CT subsystem also provides features (self-gated cardiac and respiratory imaging) and flexibility that could also be used for many dedicated CT scanner applications including skeletal and bone measurements (e.g., rat OVX osteoporosis models), cardiac and pulmonary disease, and tissue segmentation (e.g., metabolic disease) protocols.

Radiation and dose exposures reduction efforts are critical in medical/research imaging. Multiple researchers are actively engaged in the development of clinical total-body PET hardware. Human total-body PET promises improvements in dose reductions, reduced scan times, and quantitative kinetic modeling capabilities. Here, we have demonstrated that total-body PET imaging in preclinical imaging may offer many of the same benefits. Further, when combined with a low-dose CT system, dose and throughput benefits can be further enhanced.

## Ethics Statement

Experiments in Europe: all animals were kept in individual ventilated cages with *ad libitum* access to food and water. All principles of laboratory animal care were followed according to the latest European (Directive 2010/63/EU) regulations on the protection of animals used for scientific purpose and performed within the institutional guidelines. All animal experimental procedures were approved by the Ethics Committee for animal experimentation of the KU Leuven (ECD number p075/2016) and of the canton de Vaud (*Service de la consommation et des affaires vétérinaires authorization: VD2993*).

Experiments in the USA: animal experiments were approved by the Institutional Animal Care & Use Committees of the University of Virginia and performed according to the National Institutes of Health Guide for the Care and Use of Laboratory Animals.

## Author Contributions

All authors listed have made a substantial, direct and intellectual contribution to the work, and approved it for publication.

### Conflict of Interest Statement

CM, TS, CC, AB, PS, AA, SS, SJ, KL, SV, and MH are Bruker employees. The remaining authors declare that the research was conducted in the absence of any commercial or financial relationships that could be construed as a potential conflict of interest.
